# Time constant to determine PEEP levels in mechanically ventilated COVID-19 ARDS: a feasibility study

**DOI:** 10.1186/s12871-022-01935-8

**Published:** 2022-12-13

**Authors:** Filip Depta, Neil R. Euliano, Marko Zdravkovic, Pavol Török, Michael A. Gentile

**Affiliations:** 1 Department of Critical Care, East Slovak Institute for Cardiovascular Diseases, Košice, Slovakia; 2 Faculty of Medicine, Pavol Jozef Šafárik University, Košice, Slovakia; 3 Convergent Engineering, Gainesville, FL USA; 4 Department of Anaesthesiology, Intensive Care and Pain Management, University Medical Centre Maribor, Maribor, Slovenia; 5 Faculty of Medicine, University of Ljubljana, Ljubljana, Slovenia; 6 Department of Anesthesiology, Duke University Medical Center, Durham, NC USA

**Keywords:** Time constant, COVID-19, Acute respiratory distress syndrome, Positive end-expiration pressure, PEEP titration

## Abstract

**Background:**

We hypothesized that the measured expiratory time constant (TauE) could be a bedside parameter for the evaluation of positive end-expiratory pressure (PEEP) settings in mechanically ventilated COVID-19 patients during pressure-controlled ventilation (PCV).

**Methods:**

A prospective study was conducted including consecutively admitted adults (*n* = 16) with COVID-19-related ARDS requiring mechanical ventilation. A PEEP titration using PCV with a fixed driving pressure of 14 cmH_2_O was performed and TauE recorded at each PEEP level (0 to 18 cmH_2_O) in prone (*n* = 29) or supine (*n* = 24) positions. The PEEP setting with the highest TauE (TauE_MAX_) was considered to represent the best tradeoff between recruitment and overdistention.

**Results:**

Two groups of patterns were observed in the TauE plots: recruitable (R) (75%) and nonrecruitable (NR) (25%). In the R group, the optimal PEEP and PEEP ranges were 8 ± 3 cmH_2_O and 6–10 cmH_2_O for the prone position and 9 ± 3 cmH_2_O and 7–12 cmH_2_O for the supine position. In the NR group, the optimal PEEP and PEEP ranges were 4 ± 4 cmH_2_O and 1–8 cmH_2_O for the prone position and 5 ± 3 cmH_2_O and 1–7 cmH_2_O for the supine position, respectively. The R group showed significantly higher optimal PEEP (*p* < 0.004) and PEEP ranges (*p* < 0.001) than the NR group. Forty-five percent of measurements resulted in the most optimal PEEP being significantly different between the positions (*p* < 0.01). Moderate positive correlation has been found between TauE vs C_RS_ at all PEEP levels (r^2^ = 0.43, *p* < 0.001).

**Conclusions:**

TauE may be a novel method to assess PEEP levels. There was wide variation in patient responses to PEEP, which indicates the need for personalized evaluation.

## Background

Mechanical ventilation has revolutionized intensive care medicine in the twentieth century [1]. Although it can be a life-supporting intervention, it can also contribute to lung injury through stress and strain, referred to as ventilator-induced lung injury (VILI), even in previously healthy lungs [2]. The impact may be worse when significant lung nonhomogeneity is present, as with acute lung injury and acute respiratory distress syndrome (ARDS) [3]. The basic goal of protective ventilation is to preserve the function of healthy areas (prevention of alveolar overdistension) and to decrease ventilation nonhomogeneity [4]. However, in the conventional protective ventilation strategy, which combines low tidal volume (Vt) with sufficient positive end-expiratory pressure (PEEP) to keep the alveoli open, the selection of the optimal PEEP level to balance recruitment and avoid alveolar overdistension for an individual patient is still heavily debated in clinical practice [5–7].

There are various methods to determine appropriate PEEP levels (i.e. compliance of the respiratory system—C_RS_, lower inflection point, transpulmonary pressure, etc.) with most of them being assessed during inspiratory phase of the respiratory cycle [8]. In contrast to inspiratory variables, we decided to assess PEEP levels during exhalation using expiratory time constant (TauE). The main reason to propose such new method to titrate PEEP is including compliance as well as airway resistance that has been shown to change during tidal ventilation [9, 10]. To our knowledge, exhalation dynamics using TauE has not been used to assess optimal PEEP levels to date.

The TauE determines the rate of change in the volume of the lung. There is a 63% change in expiratory tidal volume (Vte) during the first TauE [11]. The TauE can be calculated as the product of respiratory system compliance (C_RS_) and airway resistance (R_AW_) and therefore changes in TauE reflect not only changes in respiratory system physiology (C_RS_ and R_AW_) but also changes in Vt [12, 13]. Increases in TauE due to increases in PEEP are likely due to recruitment (increased Vt and C_RS_), while decreases in TauE with higher PEEP levels are likely due to overstretching the alveoli. There is a complex balance between these two phenomena in nonhomogeneous lungs.

In this study, we aimed to determine optimal PEEP levels dictated by highest *measured* TauE. We also compared the optimal PEEP obtained by TauE to optimal PEEP as determined by C_RS_ [14].

## Methods

### Study design and participants

This prospective observational study was performed in a tertiary teaching hospital (East Slovak Institute for Cardiovascular Diseases, Slovakia). Approval was obtained from the Institutional Ethics Committee of East Slovak Institute of Cardiovascular Diseases, Košice, Slovakia (IEC No. EK – 01/2021). A waiver of informed consent was issued by the same ethics committee due to PEEP titration method that was considered routine clinical practice.

Consecutive patients (n = 16) admitted to the ICU from March until April 2021 and diagnosed with COVID-19 pneumonia, confirmed by polymerase chain reaction, were enrolled in the study. Patients were required to have moderate or severe ARDS according to the Berlin definition (PaO_2_:FiO_2_ ratio < 200 with PEEP > 5 cmH_2_O) [15]. As part of standard clinical care at our hospital, all patients were sedated using continuous infusion of propofol and sufentanil and received continuous neuromuscular blockade with either atracurium or rocuronium. Also, as part of routine clinical practice and as per ventilator default, the trigger sensitivity was set at 1 l/min and the ratio of triggered to mandatory (T/M) breaths displayed on the ventilator had to be zero. Therefore, if patients had any spontaneous breathing efforts shown as triggering on the ventilator graphics or the T/M was higher than zero, patients were not included to the study.

All PEEP titrations were obtained during the first 5 days of mechanical ventilation. As the study was conducted during an unprecedented strain on the healthcare system, a pragmatic approach was chosen for PEEP titrations, i.e. to perform up to 5 PEEP titrations per patient in 12 to 24 h intervals in supine and prone patient positions. Thus, in seven patients, the measurements were performed in supine and prone positions within 15 min of position change (i.e., one measurement in each position—total 14 PEEP titrations). In five patients PEEP titrations were measured repeatedly in 12–24 h interval in both positions resulting in further 26 PEEP titrations. The remaining four patients were measured in either prone or supine position at the same 12–24 h interval resulting in further 13 PEEP titrations. The total number of PEEP titrations in 16 patients in both positions was thus 53.

### Measurements of the ventilatory parameters

TauE was measured using a mechanical ventilator Aura V (Chirana Medical, Stará Turá, Slovakia). The following ventilation parameters were used for all patients: pressure-controlled ventilation (PCV), frequency of 18 breaths per minute, I:E ratio of 1:1.5, and maximal inspiratory flow of 60 L per minute. A sensor was used at the end of the tracheal tube to measure flow and pressure. Before measurements, patients were preoxygenated with 100% oxygen for 5 min. Then, an end-expiratory pause with zero end-expiratory pressure was applied for 5 s to achieve full exhalation. PEEP levels were set in the escalating order of 0, 5, 8, 10, 12, 15 and 18 cmH_2_O. An inspiratory pressure of 14 cmH_2_O was applied on top of each PEEP. TauE along with other parameters once stabilized (i.e., during the last 10 of 15 consecutive breaths at each PEEP level) were then recorded. TauE typically required only approximately 5 breaths to equilibrate after each change in PEEP level. C_RS_ was calculated as: C_RS_ = Vte / (PIP – PEEP), where PIP is peak inspiratory pressure and Vte is expiratory tidal volume [16, 17].

### TauE measurement

The first TauE is the time required for deflation of an end-inspiratory volume by 63% during passive exhalation. The mechanical ventilator measured the time required to exhale 63% of the delivered Vt from expiratory flow curve of the previous breath. Measured TauE was then displayed on the ventilator and recorded as an average of the last 10 breaths.

### Outcomes and definitions

The main goal of this study was to explore clinical feasibility of TauE to determine the optimal PEEP levels and optimal PEEP range. The optimal PEEP was defined as the PEEP level where the maximum TauE (TauE_MAX_) was found on the PEEP versus TauE plot. The optimal PEEP range was defined as the PEEP range correlating with a 5% variation from TauE_MAX_ based on the assumption to represent similar lung mechanics (TauE, Vte and C_RS_). The optimal PEEP and optimal PEEP range as determined by TauE were compared to the optimal PEEP and optimal PEEP range as determined by C_RS_. C_RS_ correlation to TauE was used for evaluation of this new PEEP titration method.

During ascending PEEP trials using constant inspiratory pressure, lungs were described as recruitable if increase in tidal volume was observed with ascending PEEP [18]. Because TauE was directly measured from Vte and the increasing Vte usually coincided with increasing TauE, recruitability patterns were determined similarly. The measured data was split into two groups, recruitable and non-recruitable. Recruitable was defined as either greater than 10% increase in TauE as PEEP increased, or TauE remained almost constant with increasing PEEP. Nonrecruitable group was defined as similar TauE values at low PEEP levels (0–8 cmH_2_O) and then decreased substantially (> 10%) as PEEPs continued to increase.

Also PEEP levels in the prone position were compared with PEEP in the supine position to evaluate any potential recruitability patterns in the PEEP versus TauE plots.

### Statistical analysis

Categorical data are expressed as number (percentage) [n (%)], continuous data are expressed as the mean ± standard deviation (SD) for normally distributed data or median with interquartile range (IQR) for nonnormally distributed data. We compared the recruitable with nonrecruitable patterns with the student’s t-test. Linear correlation analysis was performed to compare C_RS_ with TauE. Data were analyzed using statistical software (MATLAB, version R2018a, The MathWorks Inc, Natick, MA, USA).

## Results

Fifty-three PEEP titrations were performed in the prone (*n* = 29) and supine (*n* = 24) positions. Four (25%) patients had the PaO_2_:FiO_2_ ratio 100 – 200 and 12 (75%) had PaO_2_:FiO_2_ ratio < 100. Baseline patient characteristics are shown in Table 1.

**Table 1:** Baseline characteristics of patients in the first 24 h of ICU admission due to COVID-19

*Demographic data*	n = 16	
Age (years), mean (SD)		56 (12)
Male, n (%)		12 (75%)
Female, n (%)		4 (25%)
Body mass index (kg.m^−2^), mean (SD)		33 (7)
***Scoring systems on admission***		
APACHE II score, median (IQR)		13 [12–19]
SOFA score, median (IQR)		7 [5–9]
PaO_**2**_/F_I_O_2_ ratio, mean (SD)		74 (31)
***Medical History, n (%)***		
Hypertension		10 (62%)
Diabetes		8 (50%)
Chronic heart failure		5 (31%)
Chronic kidney disease		3 (18%)
Chronic obstructive pulmonary disease / Asthma		1 (6%)
Smoking		5 (30%)
Autoimmune		1 (6%)
Others ^a^		4 (25%)
***Adjunctive therapies, n (%)***		
Prone position		14 (87%)
Neuromuscular blocking agents		16 (100%)
Corticosteroids for COVID-19 ^b^		16 (100%)
Veno-venous extracorporeal membrane oxygenation		2 (12%)
Continuous renal replacement therapy		7 (43%)
***Outcomes, median (IQR)***		
Duration of mechanical ventilation (days)		11 [7,36]
ICU length of stay (days)		14 [9,47]
30-day mortality (n, %)		4 (25%)

SD – standard deviation, n – number, APACHE II – acute physiology and chronic health evaluation 2, IQR – interquartile range, SOFA score – sequential organ failure assessment score, PaO_**2**_ – partial pressure of oxygen in arterial blood. ^a^ Other includes endocrine disorders, neurologic disorders, chronic liver disease, ^b^ dexamethasone 8 mg per day while on mechanical ventilation

**Fig. 1:**
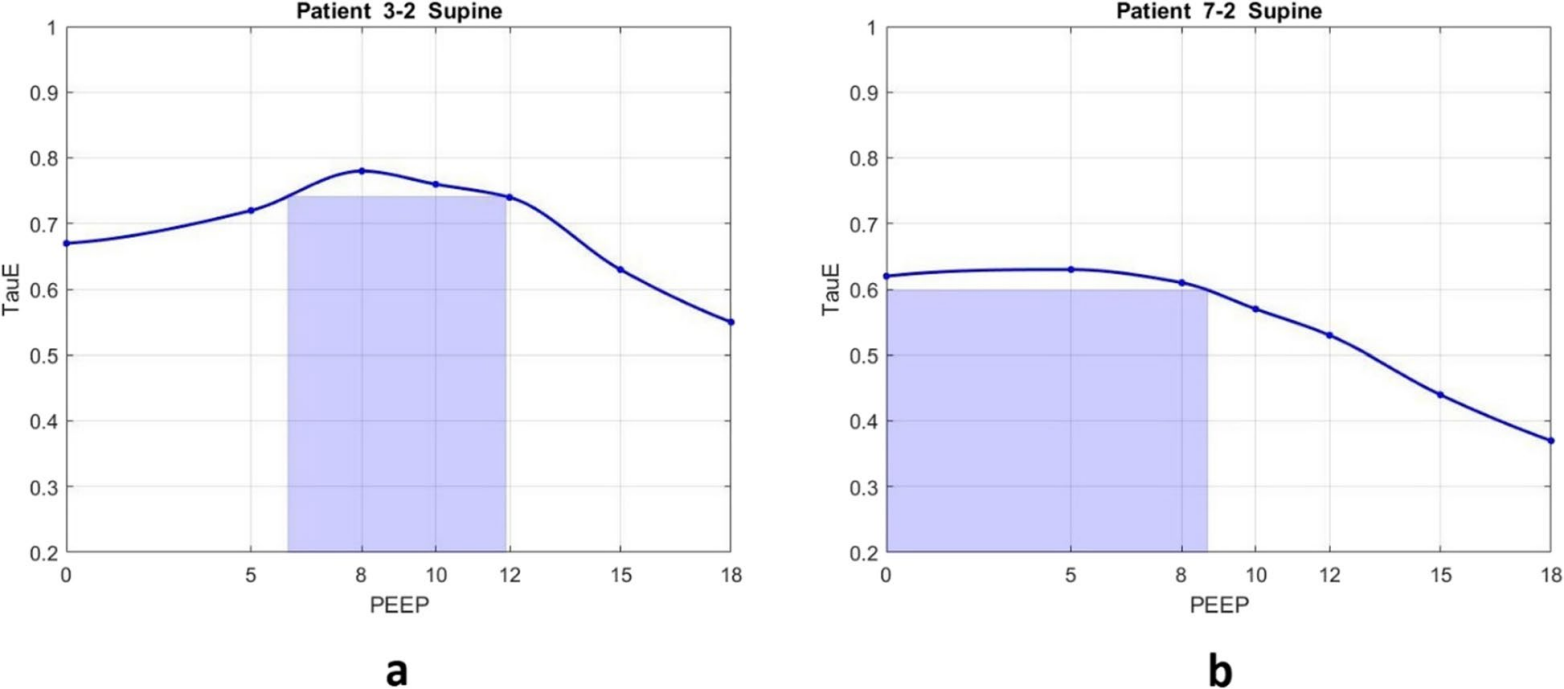
Examples of PEEP versus TauE plots showing the recruitable (a) and nonrecruitable (b) groups. In (a) the maximum TauE occurs around PEEP of 8 cmH_2_O, and the optimal PEEP range (where TauE is within 5% of its maximum value) was identified between 6 and 12 cmH_2_O. In (b), nonrecruitable pattern was found with maximum TauE at 5 cmH_2_O the optimal PEEP range was between 0 and 8 cmH_2_O, likely indicating no significant recruitment at higher PEEP levels. TauE – expiratory time constant, PEEP – positive end expiratory pressure

**Fig. 2:**
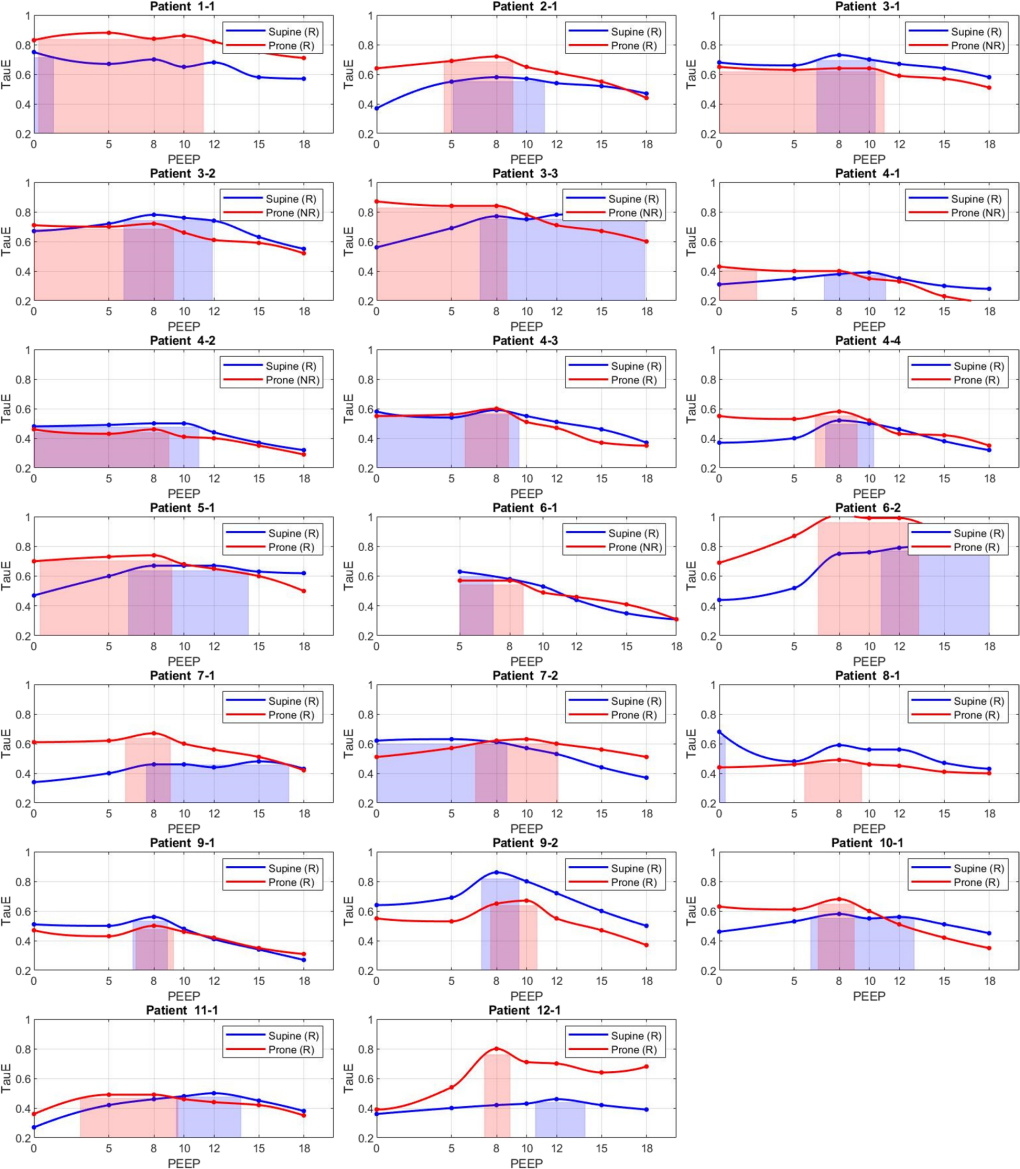
Prone versus supine plots showing optimal PEEP range (indicated by colored boxes) according to the maximum expiratory time constant (TauE) ± 5%. NR – non-recruitable pattern, R – recruitable pattern, PEEP – positive end-expiratory pressure

From the 53 PEEP versus TauE plots obtained, 40 (75%) were recruitable and 13 (25%) nonrecruitable (Table 2). PEEP values within 5% of the TauE_MAX_ were, considered to be in the ‘optimal PEEP range’ and are indicated by colored boxes on the plots (Figs. 1 and 2).

**Table 2:** Pattern groups based on positive end-expiratory pressure (PEEP) versus TauE

GROUP	DEFINITION	n (%)
RECRUITABLE	TauE shows evidence of recruitment (increase in TauE, or no significant change in TauE with increasing PEEP using constant inspiratory pressure) (Fig. 1a)	40 (75%)
NONRECRUITABLE	TauE did not show evidence of recruitment (decrease in TauE with increasing PEEP using constant inspiratory pressure) (Fig. 1b)	13 (25%)

n – number of PEEP titrations, TauE – expiratory time constant, PEEP – positive end-expiratory pressure

Measurements of optimal PEEP and optimal PEEP range using TauE are shown in Table 3. The recruitable group showed significantly higher optimal PEEP than the nonrecruitable group (*p* < 0.001). The comparison of Vte, PEEP and PEEP ranges for supine and prone positions are shown in Table 4. The optimal PEEP was higher and the Vte lower in the supine position when compared to the prone position for recruitable patterns: *p* < 0.001 and *p* = 0.26, respectively. The width of the optimal PEEP range was not significantly different (*p* = 0.09) between the two patient positions. For the non-recruitable patterns there was no difference in optimal PEEP, PEEP range and Vte between the supine and prone positions: *p* = 0.47, p = 0.82, and *p* = 0.48, respectively.

**Table 3:** Analysis of all 53 PEEP titrations using TauE

	**RECRUITABLE**	**NON-RECRUITABLE**	***p* values**
n	40	13	N/A
Vte (ml/kg/PBW)	8.4 (1.2)	8.0 (1.1)	0.29
PEEP (cmH_2_O) *	9 (3.5)	4 (3.5)	0.001
PEEP range size (cmH_2_O) *	6 – 11	1 – 7	0.091

Analysis of all 53 PEEP titrations showing the optimal PEEP and optimal PEEP range using the expiratory time constant (TauE). Standard deviations are shown in parenthesis. *optimal PEEP based on maximum TauE (TauE_MAX_) **optimal PEEP range based on maximum TauE (TauE_MAX_) ± 5%. n – number, N/A – not applicable, Vte – expiratory tidal volume, PBW – predicted body weight.

**Table 4:** Analysis of all 53 PEEP titrations using TauE in different positions

	**PRONE POSITION** n = 29		**SUPINE POSITION** n = 24	
	**Recruitable**	**Non-recruitable**	**Recruitable**	**Non-recruitable**
n	21	8	19	5
Vte (ml/kg/PBW)	9.2 (1.3)	7.9 (1.2)	7.6 (1.1)	8.4 (1.2)
PEEP (cmH_2_O) *	8 (3)	4 (4)	9 (4)	5 (3)
PEEP range size (cmH_2_O) **	6—10	1—8	7—12	1 – 7

Analysis of 53 positive end-expiratory pressure (PEEP) titrations showing the optimal PEEP and optimal PEEP range using the expiratory time constant (TauE) in different patient positions. Standard deviations are shown in parenthesis. * optimal PEEP based on the highest TauE (TauE_MAX_), ** optimal PEEP range based on TauE_MAX_ ± 5%. n – number, Vte – expiratory tidal volume, PBW – predicted body weight

### Comparison of optimal PEEP levels as determined by TauE versus compliance

For comparison to traditional methods of PEEP titration, we compared the optimal PEEP and optimal PEEP range using C_RS_ in the same way as using TauE method (Table 5). There was no difference in the mean optimal PEEP between TauE method and C_RS_ method (*p* < 0.09). However, in the recruitable group, the optimal PEEP range was significantly wider with C_RS_ than with TauE method, both for prone (*p* = 0.016) and supine (*p* = 0.02) positions. In the nonrecruitable group, the width of the PEEP range based on C_RS_ was wider but not statistically significant in the prone (*p* = 0.19) or supine positions (*p* = 0.24).

**Table 5:** Comparison of TauE vs Compliance

	**PRONE POSITION** *n* = 29		**SUPINE POSITION** *n* = 24	
	**Recruitable**	**Non-recruitable**	**Recruitable**	**Non-recruitable**
n	21	8	19	5
PEEP TauE (cmH_2_O)	8 (3)	4 (4)	9 (4)	5 (3)
PEEP C_RS_ (cmH_2_O)	7 (2)	5 (4)	11 (4)	5 (2)
PEEP Range TauE (cmH_2_O)	6—10	1—8	7—12	1 – 7
PEEP Range C_RS_ (cmH_2_O)	5—14	0—11	6—15	0—10

Comparison of the optimal positive end-expiratory pressure (PEEP) and optimal PEEP range using the maximum expiratory time constant (TauE) or the highest compliance (CRS). Optimal PEEP range was identified as maximum TauE (TauEMAX) ± 5% or maximum CRS ± 5%. Standard deviations are shown in parenthesis. n – number, PEEP – positive end-expiratory pressure, TauEMAX – maximum expiratory time constant, CRS – dynamic compliance

**Fig. 3:**
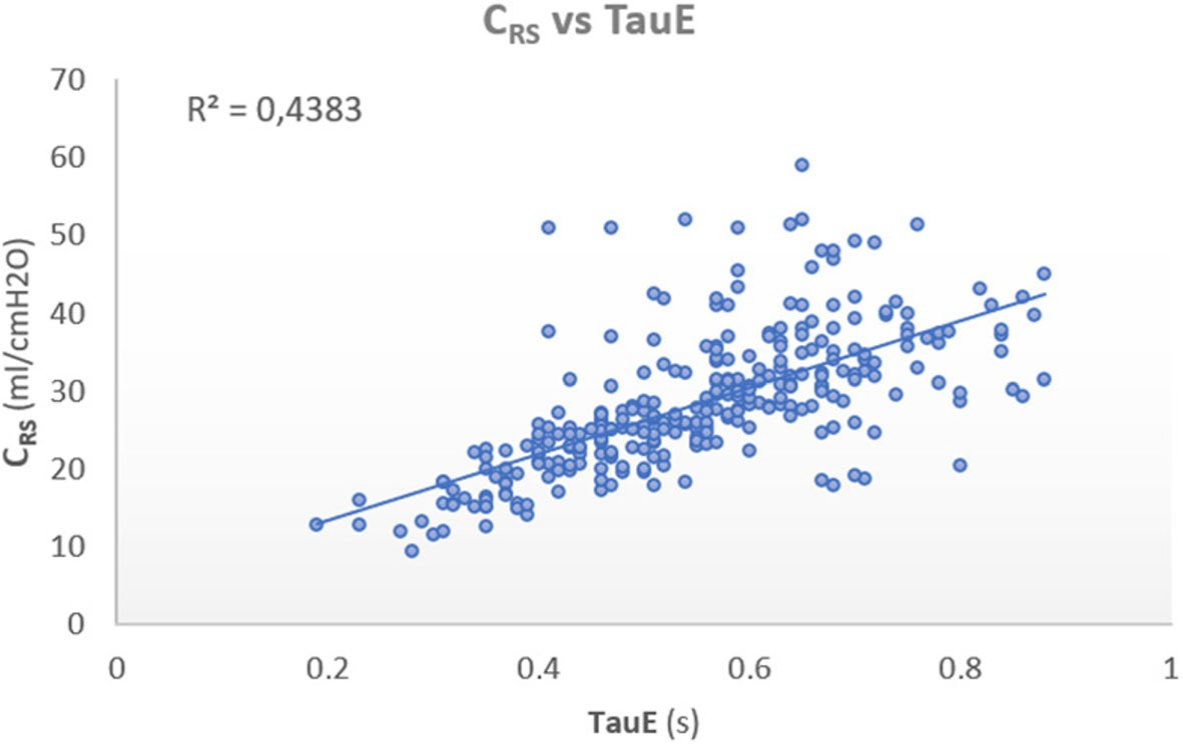
Linear correlation plot showing compliance of the respiratory system (C_RS_) versus expiratory time constant (TauE) at all PEEP levels (r^2^ = 0.43, p < 0.001)

We also correlated the C_RS_ with TauE at all PEEP levels (Fig. 3) showing moderate positive correlation (r^2^ = 0.43, *p* < 0.001 for TauE vs C_RS_). However, we also found there was high individual variation when assessing optimal PEEP using TauE and optimal PEEP using C_RS_.

## Discussion

The main finding of this study is that TauE may be novel and feasible method to assess the physiologic responses to changes in PEEP in patients with COVID-19 related ARDS. The mean values of obtained optimal PEEP levels were very similar to the one determined by the C_RS_ but the overall correlation of TauE with C_RS_ was only moderate which suggests the need for personalization.

Similar to other parameters (i.e. C_RS_, VdVt, oxygenation), TauE may be used to distinguish between the recruitable and non-recruitable lungs when PEEP titration is performed. We suggested that expiratory pulmonary mechanics, rather than conventional inspiratory lung mechanics, can be used to examine responses to PEEP levels. Also, in the supine position there is a higher optimal PEEP when compared to the prone position and a smaller Vte. This is in line with previous findings on ARDS ventilation differences between the prone and supine positions [19, 20].

TauE was selected to be evaluated in this study as it may have additional advantages over other variables of recruitment due to the following reasons: first, exhalation time reflects dynamics (time for which 63% of Vt is exhaled) instead of static parameters routinely used to assess pulmonary function (i.e. C_RS_ or driving pressure); second, TauE reflects the speed of elastic recoil which may reflect overdistension more sensitively; third, TauE may also reflects a change in airways resistance during exhalation that will subsequently change the time constant [9]**.** More, the mean airway pressure is still high during early exhalation to allow continuing Vt equilibration among different time constant regions in nonhomogenous lungs and such aspect may also be reflected in average measured TauE of the whole respiratory system. Most of these aspects are not accounted for in static inspiratory lung parameters that are routinely used for assessing recruitment [21].

Some mechanical ventilators provide a calculated TauE value whereas some measure the TauE directly. It has been shown that calculated and measured time constant differ [22]. In contrast to calculated time constants (TauE = R_AW_ * C_RS_), measured TauE reflects changes in the whole respiratory system, including artificial airways, breathing circuits, humidification devices and mechanical ventilator [22].

Mechanical ventilation practices vary widely amongst institutions. Setting up the “optimal” PEEP has been a subject of vigorous debate in the scientific community [23]. It can be said that potential complications to using inappropriate PEEP levels is either alveolar overdistension or cyclic alveolar collapse. However, cyclic collapse of the airways during mechanical ventilation remains controversial suggesting heterogenous distension rather than collapse [24, 25]. Further studies should therefore focus on assessing cyclic alveolar collapse during ventilation using multiple alternating levels of PEEP [26].

Additionally, two potential patterns of lung recruitability based on the PEEP versus the TauE plots may be identified. It can be assumed during PEEP titration, that patterns where TauE increases and then decreases represent recruitment followed by overdistension. The non-recruitable patterns tend to descend throughout and show lungs that cannot be recruited even at high PEEP levels. Using these patterns, various degrees of baby lungs may potentially be identified. With the nonrecruitable pattern, we assume that higher PEEP levels will be harmful due to overdistention of relatively healthy areas of the ‘baby lung’, as there is no evidence of recruitability.

The critical care community is recognizing the need for personalized medicine. Applied physiology and assessing real measured time constants could comprise another approach to personalized mechanical ventilation for each individual patient [27]. From the PEEP versus TauE plots constructed in the sequential study of the same patients, pulmonary mechanics shifted as ARDS changed over time (i.e. differences in TauE patterns, recruitability, optimal PEEP and PEEP ranges were observed in time). Therefore, optimization of ventilation and PEEP may be required more frequently than previously thought, and PEEP versus TauE plots may be an efficient method of assessing these changes in real time at the bedside.

This study has several limitations. Sample size included only a relatively small group of patients who presented with moderate to severe COVID-19 related ARDS. The findings might not be generalizable to other causes of ARDS and more rigorous study designs are needed to confirm our findings. Another possible limitation is that the equilibration time needed for proper recruitment at each PEEP level varies from patient to patient. Our study was designed to obtain results practically and quickly at bedside due to critical illness associated with hypoxemia in ARDS patients. Thus, TauE at each PEEP level was evaluated during 15 breaths only. Time spent at each PEEP could have therefore been relatively short for recruitment to manifest fully.

What is more, because PCV was used without an end-expiratory pause, plateau pressures used to calculate static compliance were not obtained. For that reason, dynamic compliance was used. Dynamic compliance might underestimate true (static) compliance due to the resistive pressure, but as reported in previous studies that also used dynamic compliance during descending PEEP trial, correlation between dynamic and static compliance was very high (*r* = 0.92) [16]. More, Stahl suggested that application of dynamic respiratory mechanics as a diagnostic tool in ventilated patients should be more appropriate than using static pressure–volume curves [17].

Also, because the study was conducted during collapsing healthcare system during COVID-19 pandemic, strict adherence to the measurement protocol could not be guaranteed. Therefore, pragmatic approach was chosen for PEEP titrations that usually resulted in variable PEEP titration count per patient.

Expiratory lung mechanics described by TauE may be a promising approach for PEEP titration in ARDS. Several further studies are warranted in this field due to the scarcity of publications on time constant in clinical practice. Comparative, randomised studies of inspiratory versus expiratory lung mechanics for PEEP titration are needed. Imaging techniques might be used for the comparison of the two strategies as well as for objectivizing recruitment obtained using TauE. To demonstrate its potential benefit versus compliance method for PEEP titration in terms of less VILI and studying the mediators of VILI would be very interesting [28, 29]. Also, some laboratory studies on animals with ARDS and benchmark studies on TauE in different pulmonary pathology by using the test lungs would provide further insight into the expiratory pulmonary mechanics and their role in finding optimal PEEP for lung recruitment.

## Conclusions

Expiratory time constant may represent a feasible method to assess the physiologic responses to changes in PEEP and may be a promising approach for PEEP titration. Assessing real measured time constants could comprise another approach to personalized mechanical ventilation for each individual patient. Repeated measurements are likely beneficial, and personalized optimization of ventilation should be done frequently during the initial stages of ARDS to ensure the most protective ventilation. Additional clinical studies evaluating recruitment and the usefulness of TauE are warranted to assess utility and validity in ARDS due to different etiologies.

## Data Availability

Any data-related questions should be directed to the corresponding author.

## Abbreviations

**APACHE II score**: Acute physiology and chronic health evaluation 2 score
**ARDS**: Acute respiratory distress syndrome
**COVID-19**: Coronavirus disease 2019
**C**_**RS**_: Respiratory system compliance
**FiO**_**2**_: Fraction of inspired oxygen
**ICU**: Intensive care unit
I**:E ratio**: inspiratory to expiratory ratio
**IQR**: Interquartile range
**PaO**_**2**_: Partial pressure of oxygen in the arterial blood
**PBW**: Predicted body weight
**PCV**: Pressure-controlled ventilation
**PEEP**: Positive end-expiratory pressure
**PIP**: Peak inspiratory pressure
**R**_**AW**_: Airway resistance
**SD**: Standard deviation
**SOFA score**: Sequential organ failure assessment score
**SpO2**: oxygen saturation using pulse oximetry
**TauE**: First expiratory time constant
**TauE**_**MAX**_: Maximum TauE
**VILI**: Ventilator induced lung injury
**Vt**: Tidal volume
**Vte**: Expiratory tidal volume

## References

[1] MacIntyre N, Rackley C, Khusid F (2021). Fifty Years of Mechanical Ventilation-1970s to 2020. Crit Care Med.

[2] Pelosi P, Rocco PR (2011). Ventilator-induced lung injury in healthy and diseased lungs: better to prevent than cure!. Anesthesiology.

[3] Beitler JR, Malhotra A, Thompson BT (2016). Ventilator-induced Lung Injury. Clin Chest Med.

[4] Gattinoni L, Tonetti T, Quintel M (2017). Regional physiology of ARDS. Crit Care.

[5] Cressoni M, Chiumello D, Algieri I (2017). Opening pressures and atelectrauma in acute respiratory distress syndrome. Intensive Care Med.

[6] The Acute Respiratory Distress Syndrome Network Ventilation with lower tidal volumes as compared with traditional tidal volumes for acute lung injury and the acute respiratory distress syndrome. N Engl J Med. 2000;**342**:1301–1308. 10.1056/NEJM200005043421801 10793162

[7] Marini JJ (2019). How I optimize power to avoid VILI. Crit Care.

[8] Silva PL, Rocco PRM (2018). The basics of respiratory mechanics: ventilator-derived parameters. Ann Transl Med.

[9] Guérin C, Fournier G, Milic-Emili J (2001). Effects of PEEP on inspiratory resistance in mechanically ventilated COPD patients. Eur Respir J.

[10] Smith TC, Marini JJ. Impact of PEEP on lung mechanics and work of breathing in severe airflow obstruction. J Appl Physiol (1985). 1988;65(4):1488–1499 10.1152/jappl.1988.65.4.1488 3053583

[11] Hess DR (2014). Respiratory mechanics in mechanically ventilated patients. Respir Care.

[12] Shevade MS (2019). Time constant: What do we need to know to use it?. Indian J Respir Care.

[13] Al-Rawas N, Banner MJ, Euliano NR, et al. Expiratory time constant for determinations of plateau pressure, respiratory system compliance, and total resistance. Crit Care. 2013;**17**(1):R23. Published 2013 10.1186/cc12500 PMC4056774 23384402

[14] Sahetya SK, Goligher EC, Brower RG. Fifty Years of Research in ARDS. Setting Positive End-Expiratory Pressure in Acute Respiratory Distress Syndrome. Am J Respir Crit Care Med. 2017;**195**(11):1429–14385 10.1164/rccm.201610-2035CI PMC5470753 28146639

[15] Definition Task Force ARDS, Ranieri VM, Rubenfeld GD, Thompson BT, Ferguson ND et al. Acute respiratory distress syndrome: the Berlin definition. JAMA. 2012;**307**(23):2526–2533 10.1001/jama.2012.5669 22797452

[16] Suarez-Sipmann F, Böhm SH, Tusman G, Pesch T, Thamm O, Reissmann H, Reske A, Magnusson A, Hedenstierna G (2007). Use of dynamic compliance for open lung positive end-expiratory pressure titration in an experimental study. Crit Care Med.

[17] Stahl CA, Möller K, Schumann S (2006). Dynamic versus static respiratory mechanics in acute lung injury and acute respiratory distress syndrome. Crit Care Med.

[18] Del Sorbo L, Tonetti T, Ranieri VM (2019). Alveolar recruitment in acute respiratory distress syndrome: should we open the lung (no matter what) or may accept (part of) the lung closed?. Intensive Care Med.

[19] Langer T, Brioni M, Guzzardella A (2021). Prone position in intubated, mechanically ventilated patients with COVID-19: a multi-centric study of more than 1000 patients. Crit Care.

[20] Park, J., Lee, H.Y., Lee, J. et al. Effect of prone positioning on oxygenation and static respiratory system compliance in COVID-19 ARDS vs. non-COVID ARDS. Respir Res 22, 220 (2021) 10.1186/s12931-021-01819-4 PMC8343350 34362368

[21] Caramez MP, Kacmarek RM, Helmy M (2009). A comparison of methods to identify open-lung PEEP. Intensive Care Med.

[22] Guttmann J, Eberhard L, Fabry B (1995). Time constant/volume relationship of passive expiration in mechanically ventilated ARDS patients. Eur Respir J.

[23] Gattinoni L, Collino F, Maiolo G (2017). Positive end-expiratory pressure: how to set it at the individual level. Ann Transl Med.

[24] Grune J, Tabuchi A, Kuebler WM (2019). Alveolar dynamics during mechanical ventilation in the healthy and injured lung. ICMx.

[25] Mertens M, Tabuchi A, Meissner S, Krueger A, Schirrmann K, Kertzscher U, Pries AR, Slutsky AS, Koch E, Kuebler WM (2009). Alveolar dynamics in acute lung injury: heterogeneous distension rather than cyclic opening and collapse. Crit Care Med.

[26] Depta F, Zdravkovic M, Gentile MA (2022). Should we continue searching for the single best PEEP?. ICMx.

[27] Pelosi P, Ball L, Barbas CSV (2021). Personalized mechanical ventilation in acute respiratory distress syndrome. Crit Care.

[28] Grazioli S, Dunn-Siegrist I, Pauchard LA, Blot M, Charles PE (2019). Mitochondrial alarmins are tissue mediators of ventilator-induced lung injury and ARDS. PLoS ONE.

[29] Wallace MJ, Probyn ME, Zahra VA (2009). Early biomarkers and potential mediators of ventilation-induced lung injury in very preterm lambs. Respir Res.

